# A magnetically retrievable mixed-valent Fe_3_O_4_@SiO_2_/Pd^0^/Pd^II^ nanocomposite exhibiting facile tandem Suzuki coupling/transfer hydrogenation reaction

**DOI:** 10.1038/s41598-021-88528-6

**Published:** 2021-04-29

**Authors:** Parminder Singh, Saumyaranjan Mishra, Anupam Sahoo, Srikanta Patra

**Affiliations:** School of Basic Sciences, Indian Institute of Technology Bhubaneswar, Argul, Jatni, Odisha 752050 India

**Keywords:** Catalysis, Materials chemistry

## Abstract

Herein, we report a magnetically retrievable mixed-valent Fe_3_O_4_@SiO_2_/Pd^0^/Pd^II^NP (**5**) nanocomposite system for tandem Suzuki coupling/transfer hydrogenation reaction. The nanocomposite **5** was prepared first by making a layer of $\hbox {SiO}_{2}$ on $\hbox {Fe}_{3}\hbox {O}_{4}\hbox {NP}$ followed by deposition of $\hbox {Pd}^{0}$ and sorption of $\hbox {Pd}^{\mathrm{II}}$ ions successively onto the surface of Fe_3_O_4_@SiO_2_NP. The nanocomposite was characterized by powder XRD, electron microscopy (SEM-EDS and TEM-EDS) and XPS spectroscopy techniques. The mixed-valent $\hbox {Pd}^{0}/\hbox {Pd}^{\mathrm{II}}$ present onto the surface of nanocomposite **5** was confirmed by XPS technique. Interestingly, the mixed-valent nanocomposite Fe_3_O_4_@SiO_2_/Pd^0^/Pd^II^NP (**5**) exhibited tandem Suzuki coupling/transfer hydrogenation reaction during the reaction of aryl bromide with aryl boronic acid (90% of **C**). The nanocomposite **5** displayed much better reactivity as compared to the monovalent Fe_3_O_4_@SiO_2_/Pd^0^NP (**3**) (25% of **C**) and Fe_3_O_4_@SiO_2_/Pd^II^NP (**4**) (15% of **C**) nanocomposites. Further, because of the presence of magnetic $\hbox {Fe}_{3}\hbox {O}_{4}$, the nanocomposite displayed its facile separation from the reaction mixture and reused at least for five catalytic cycles.

## Introduction

The design and development of environmentally benign efficient catalytic systems which can conduct multiple mechanistically distinct reaction (tandem reaction) in one-pot is a fascinating area of contemporary research^1,2^. Such catalytic systems offer a greener way by reducing reaction steps, cost of the process, and most importantly reduce the generation of waste during a reaction^1,2^. The most common and successful strategy to conduct tandem reactions is to combine multiple mechanically distinct catalytic centers in one-pot under a suitable reaction condition. A major disadvantage of this strategy is catalytic incompatibility which reduces tandem efficiency^3–5^. The alternative is to incorporate different catalytic units into a single molecular framework^6–10^ or integrate into a suitable solid matrix^4,11–13^. In both cases, a well-defined ligand-framework is required to achieve desired tandem output. Adding two different metal centers into a single molecular framework/suitable solid matrix is challenging and cumbersome. Moreover, separation of catalysts after completion of the reaction and their reuse is also a concern.

Both Suzuki–Miyaura C–C coupling reaction^14^ and transfer hydrogenation reaction^15–17^ have become an indispensable tool in organic synthesis and pharmaceutical chemistry as they offer access of facile C–C bond formation and hydrogenation without use of hazardous hydrogen gas. A large number of efficient catalytic systems (both homogeneous and heterogeneous) have been developed for conducting C–C coupling reaction and transfer hydrogenation reaction. Many of the catalytic systems are used in industrial processes and are commercially available. There are many important biaryl organic motifs, crucial for the preparation of natural products and chiral pharmaceuticals, which requires both C–C coupling as well as transfer hydrogenation reaction steps^4,11,12^. A catalyst system capable of conducting both mechanistically distinct C–C coupling reaction and transfer hydrogenation reactions in one-pot is highly appealing. Nevertheless, catalyst systems capable conducting tandem Suzuki coupling/transfer hydrogenation reaction are limited^4,10–12^. In most of the cases, heterobimetallic systems (Pd–Ru) have been used as catalysts for conducting aforesaid tandem reaction^6–9,18^. Interestingly, recently our group has observed that the tandem Suzuki coupling/transfer hydrogenation reaction can be conducted using bimetallic $\hbox {Pd}^{\mathrm{II}}{-}\hbox {Pd}^{\mathrm{II}}$ system efficiently.^19^ The palladium center in its zero-oxidation state ($\hbox {Pd}^{0}$) initiates the C–C coupling reaction, whereas the palladium center in +2 oxidation state ($\hbox {Pd}^{\mathrm{II}}$) carried out transfer hydrogenation reaction. It is to be noted that in all the cases, a well-defined ligand-framework is used to make the catalysts. It will be highly attractive if such kind of catalyst systems can be made without ligand-framework. Another interesting feature of a great catalytic system is its separability and reusability. This allows the catalyst to be separated and reused after catalytic conversion and thereby enhances the catalytic efficiency. Thus, designing a catalyst system without the use of ligand with facile separation ability is highly appealing.

With their high surface to volume ratio, interesting redox, optical, and catalytic properties, nanoparticles are attractive and become a fascinating tool for the development of tailor-made materials. The nanoparticles extend the easy incorporation of second nanoparticles onto the first one and also allow facile surface modification using different functionalities. This way, different interesting properties can easily be inserted into a single nanoparticle and thereby achieving materials with advanced features and desired properties. Considering this, several multimetallic nanoparticles systems have been developed and studied. However, the study of catalytic tandem reactivity is relatively less^20–24^.

**Figure 1:**
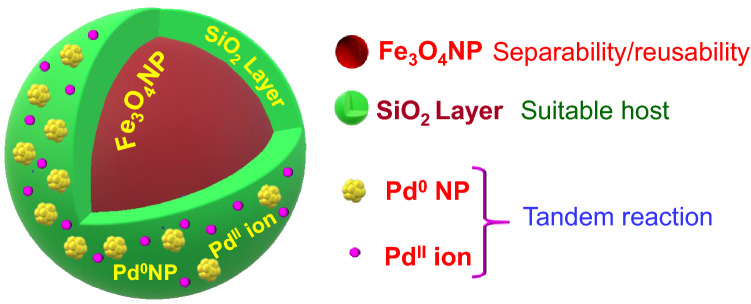
Magnetically retrievable Fe_3_O_4_@SiO_2_/Pd^0^/Pd^II^NP (**5**) nanocatalyst system and its advantages.

Utilizing the benefits of nanoparticles and advantages of one-pot multistep reactions, in the present contribution, we wish to demonstrate a magnetically retrievable mixed-valent multimetallic Fe_3_O_4_@SiO_2_/Pd^0^/Pd^II^NP (**5**) nanocatalytic system (Fig. 1) for efficient tandem Suzuki coupling/transfer hydrogenation reaction. $\hbox {Fe}_{3}\hbox {O}_{4}\hbox {NP}$ is used to insert magnetic property, and a layer of $\hbox {SiO}_{2}$ on $\hbox {Fe}_{3}\hbox {O}_{4}\hbox {NP}$ helps to host PdNP as well as $\hbox {Pd}^{\mathrm{II}}$ ions into the nanocomposite without use of any ligands. The nanocatalytic system **5** is characterized by SEM, TEM, powder XRD and XPS techniques. The catalytic reactivity of the synthesized nanocatalytic system is studied by conducting tandem Suzuki coupling/transfer hydrogenation reaction using aryl boronic acid and aryl bromide. Further, to compare the performance of the mixed-valent Fe_3_O_4_@SiO_2_/Pd^0^/Pd^II^NP (**5**) system to the monovalent Fe_3_O_4_@SiO_2_/Pd^0^NP (**3**) and Fe_3_O_4_@SiO_2_/Pd^II^NP (**4**) were also synthesized and their tandem reactivity was studied.

## Results and discussion

The magnetic $\hbox {Fe}_{3}\hbox {O}_{4}$ nanoparticles was synthesized by following the procedure reported elsewhere^25^. Briefly, a mixture of $\hbox {FeSO}_{4}$ and $\hbox {Fe}_{2}\hbox {(SO}_{4})_{3}$ (1:1 mole ratio) in alkaline solution (pH = 10) was heated at $50\,^{\circ}$C for 1 h to yield $\hbox {Fe}_{3}\hbox {O}_{4}\hbox {NP}$ (**1**). A layer of $\hbox {SiO}_{2}$ was incorporated over $\hbox {Fe}_{3}\hbox {O}_{4}\hbox {NP}$ to form Fe_3_O_4_@SiO_2_NP (**2**) by adding tetraethylorthosilicate (TEOS) to it and stirring for 18 h^25^. Palladium nanoparticle was deposited onto the surface of Fe_3_O_4_@SiO_2_NP in situ by reducing $\hbox {K}_{2}[\hbox {PdCl}_{4}]$ using $\hbox {NaBH}_{4}$ at low temperature Fe_3_O_4_@SiO_2_/Pd^0^NP (**3**)^26^. Palladium ions ($\hbox {Pd}^{\mathrm{II}}$) on the surface of Fe_3_O_4_@SiO_2_NP was sorbed by adding $\hbox {K}_{2}\hbox {PdCl}_{4}$ solution with stirring to yield $\hbox {Fe}_{3}\hbox {O}_{4}\hbox {@SiO}_{2}/\hbox {Pd}^{\mathrm{II}}$ NP (**4**). Mixed-valent Fe_3_O_4_@SiO_2_/Pd^0^/Pd^II^NP (**5**) was achieved by mixing Fe_3_O_4_@SiO_2_/Pd^0^NP and $\hbox {K}_{2}\hbox {PdCl}_{4}$ solution. All the nanoparticles were separated magnetically, followed by successive washing with triple distilled water and dried under oven for further use. A layout for the preparation of mixed-valent Fe_3_O_4_@SiO_2_/Pd^0^/Pd^II^NP (**5**) is shown in Scheme S1.

The crystalline nature of the nanoparticles was examined by powder X-ray diffraction (XRD) study. Figure S1 represents the powder XRD pattern of the nanoparticles. The powder XRD pattern of $\hbox {Fe}_{3}\hbox {O}_{4}\hbox {NP}$ (**1**) displayed peaks centered at 2 $(^{{\circ }})$ values = 30.8, 36.3, 43.9, 54.3 (s), 57.9, 63.5 and 75 (s) which could be indexed as (220), (311), (400), (422) and (511) plane corresponding to cubic lattice of $\hbox {Fe}_{3}\hbox {O}_{4}\hbox {NP}$ (**1**)[JCPDS File No: 19-0629]^27–30^. A broad band centered at $2{\uptheta }=25^{\circ }$ along with the bands for $\hbox {Fe}_{3}\hbox {O}_{4}\hbox {NP}$ was observed while a layer of silica over $\hbox {Fe}_{3}\hbox {O}_{4}$ introduced $\hbox {Fe}_{3}\hbox {O}_{4}\hbox {@SiO}_{2}\hbox {NP}$ (**2**)^29^. No significant change other than a new but very weak signal at $2= 40.27^{{\circ }}$ for $\hbox {Fe}_{3}\hbox {O}_{4}\hbox {@SiO}_{2}/\hbox {PdNP}$ (**3**) was observed while ${\hbox {Pd}}^{0}$ was deposited onto $\hbox {Fe}_{3}\hbox {O}_{4}\hbox {@SiO}_{2}\hbox {NP}$^31^. No change for Fe_3_O_4_@SiO_2_/Pd^0^/Pd^II^NP (**4**) was observed during deposition of $\hbox {Pd}^{\mathrm{II}}$ ion to $\hbox {Fe}_{3}\hbox {O}_{4}\hbox {@SiO}_{2}\hbox {NP}$. No additional peak was observed for Fe_3_O_4_@SiO_2_/Pd^0^/Pd^II^NP (**5**) during the deposition of $\hbox {Pd}^{\mathrm{II}}$ ion onto $\hbox {Fe}_{3}\hbox {O}_{4}\hbox {@SiO}_{2}\hbox {@Pd}^{0}\hbox {NP}$.

The surface morphology of the nanoparticles was investigated by Field Emission Scanning Electron Microscopy (FE-SEM) technique. The representative images are presented in Figures S2 and S3. It is observed from the images that the particles sizes are uniform and varying in the range $\sim 30 {-} 45\,\hbox {nm}$. After deposition of $\hbox {Pd}^{0}$ onto $\hbox {Fe}_{3}\hbox {O}_{4}\hbox {@SiO}_{2}\hbox {NP}$ (**2**), the $\hbox {Fe}_{3}\hbox {O}_{4}\hbox {@SiO}_{2}/\hbox {Pd}^{0}\hbox {NP}$ (**3**) gets aggregated with particle size $38.4 \pm 4.5$ nm. No significant changes were observed in the morphology while the incorporation of $\hbox {Pd}^{\mathrm{II}}$ ions $\hbox {Fe}_{3}\hbox {O}_{4}\hbox {@SiO}_{2}/\hbox {Pd}^{0}\hbox {NP}$ (**4**) (particle size $35.5 \pm 5.6$). The morphology of Fe_3_O_4_@SiO_2_/Pd^0^/Pd^II^NP (**5**) (particle size $40.4 \pm 5.4$) remains the same after the introduction of $\hbox {Pd}^{\mathrm{II}}$ ions onto $\hbox {Fe}_{3}\hbox {O}_{4}\hbox {@SiO}_{2}/\hbox {Pd}^{0}\hbox {NP}$ (**3**). The observed peaks corresponding to iron, silicon and palladium in the EDS spectra, indicating their presence in the nanocomposites (Figures S2 and S3). The elemental mapping of Fe_3_O_4_@SiO_2_/Pd^0^/Pd^II^NP (**5**) further supports the presence of the elements in the nanocomposite (Figure S3).

**Figure 2:**
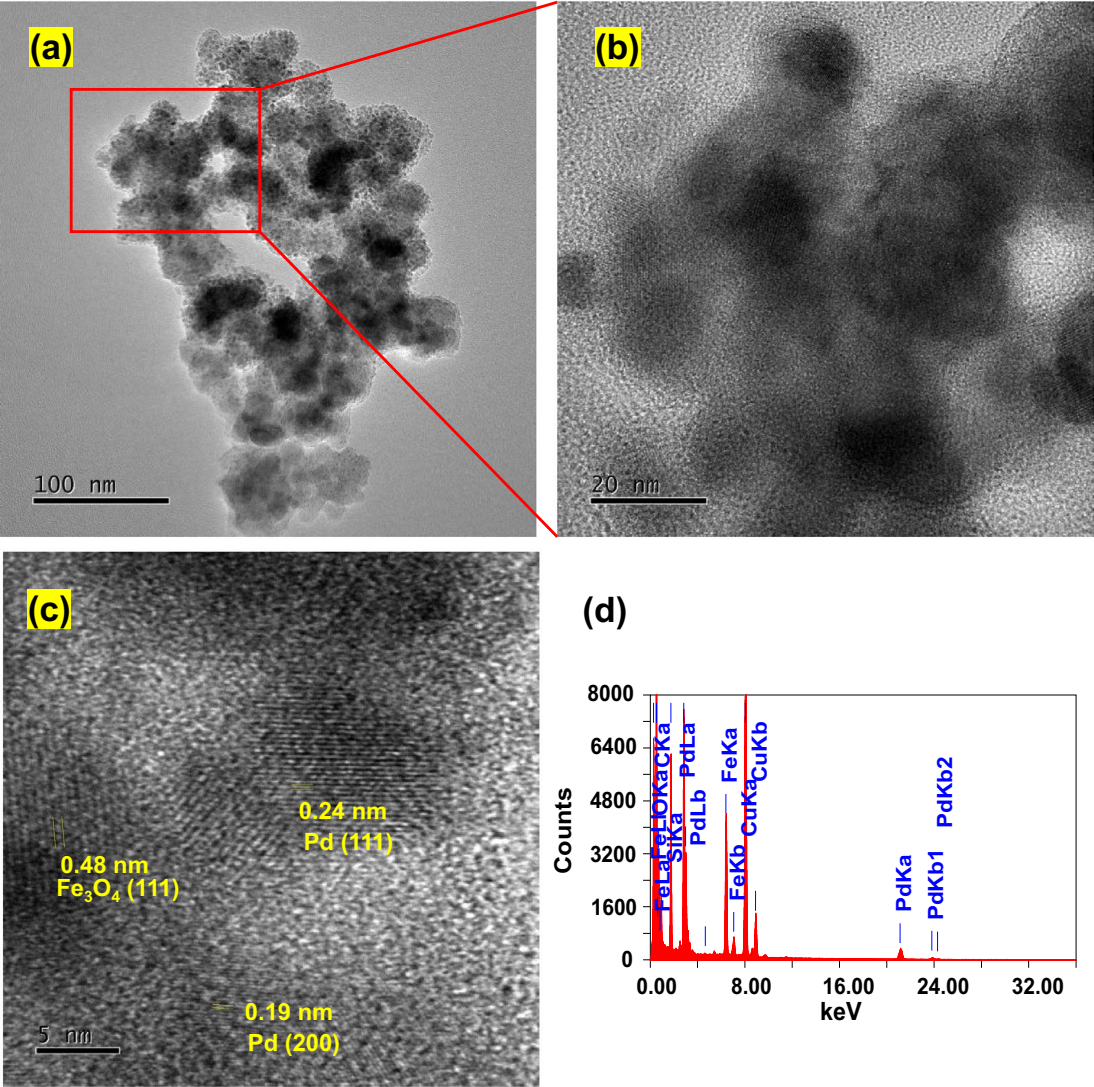
TEM and HRTEM images of Fe_3_O_4_@SiO_2_/Pd^0^/Pd^II^NP (**5**) nanocomposite.

The formation of Fe_3_O_4_@SiO_2_/Pd^0^/Pd^II^NP (**5**) was further confirmed by high-resolution transmission images microscopy (HRTEM) (Fig. 2a,b). The EDS analysis of **5** shown the peaks corresponding to iron, palladium and silicon, which are in line with SEM analysis (*vide supra*) (Fig. 2d). The HRTEM image of the nanocomposite **5** has also shown the lattice fringes 0.48 nm for oxidized iron (020)^32^, and 0.19 nm for palladium (200)^33,34^ and 0.224 nm for palladium (111)^34,35^ which further confirms the presence of the elements (Fig. 2c).

**Figure 3:**
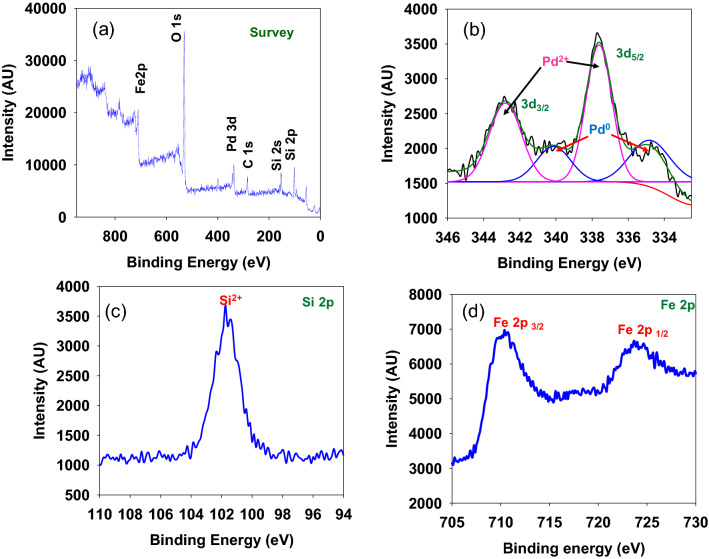
(**a**) XPS survey scan spectrum and high-resolution (**b**) Pd $\hbox {3d}_{5/2}$ and $\hbox {3d}_{3/2}$, (**c**) Si 2p and (**d**) Fe $\hbox {2p}_{3/2}$ and $\hbox {2p}_{\frac {1}{2}}$ of Fe_3_O_4_@SiO_2_/Pd^0^/Pd^II^NP (**5**).

To elucidate the surface composition and oxidation state of the elements present in the nanocomposite **5**, X-ray photoelectron spectroscopy (XPS) study was conducted (Fig. 3). The XPS survey scan spectrum of **5** discloses the presence of iron, silicon, and palladium (Fig. 3a). The high resolution XPS spectra of **5** display peaks at 101.8 eV corresponding to $\hbox {Si}^{2+}$ (Fig. 3c)^36^; 710.26 eV and 723.85 eV corresponding to $2\hbox {P}_{3/2}$ and $2\hbox {p}_{1/2}$ of $\hbox {Fe}^{2+}$ and $\hbox {Fe}^{3+}$ (Fig. 3d)^37,38^. The peaks at 334.78 eV and 340.16 eV; 337.73 eV and 342.85 eV correspond to $3\hbox {d}_{5/2}$ and $3\hbox {d}_{3/2}$ of metallic palladium ($\hbox {Pd}_{0}$) and oxidized palladium [$\hbox {Pd}^{\mathrm{II}}$: PdO or $\hbox {Pd}(\hbox {OH}_{2})_{2}]$ (Fig. 3b)^26,31,33,39–46^. This confirms presence of mixed-valent palladium ($\hbox {Pd}^{0}$ and $\hbox {Pd}^{\mathrm{II}}$) in nanocomposite **5**.

It is well-known that both $\hbox {Pd}^{0}$ and $\hbox {Pd}^{\mathrm{II}}$ can conduct efficient Suzuki coupling reaction^19,40,47–49^. Recent literatures also demonstrate that palladium (both $\hbox {Pd}^{0}$ and $\hbox {Pd}^{\mathrm{II}}$) can catalyze transfer hydrogenation reaction^19,50–57^. We, thus, presumed that the nanocatalyst Fe_3_O_4_@SiO_2_/Pd^0^/Pd^II^NP (**5**) can conduct mechanically distinct Suzuki coupling and transfer hydrogenation reactions as it comprises palladium in both $\hbox {Pd}^{\mathrm{II}}$ as well as $\hbox {Pd}^{0}$ oxidation state. With this hypothesis, we attempted to conduct tandem Suzuki coupling/transfer hydrogenation reactions. The tandem reaction was carried out using *p*-bromoacetophenone and phenylboronic acid in $^{i}\hbox {PrOH}$ as solvent and NaOH as base (Table 1). It was observed that the nanocatalyst **5** resulted in excellent yield of tandem product (entry 1, Table 1). The tandem reaction using nanocatalyst **5** was tested by varying solvents (Table 1). It was observed that the $^{i}\hbox {PrOH}$ is the best solvent for tandem reaction (entries 1, 5 and 6, Table 1). Further, the tandem reaction was found to be highly dependent on selection of base. The formation of tandem product **C** (90 %) was best during the use of NaOH as base (entry 1, Table 1). It was 85% under same reaction condition while KOH was used (entry 2, Table 1). The formation of tandem product (**C)** was significantly reduced (56%) when $\hbox {K}^{t}\hbox {OBu}$ was used as base (entry 3, Table 1). Surprisingly, no tandem product (**C**) was formed when the base $\hbox {Cs}_{2}\hbox {CO}_{3}$ was used (entry 4, Table 1), however, the yield of coupling product (**B**) was appreciably high (93%) (entry 4, Table 1). This suggests that the use of bases like KOH and NaOH plays an important role to result in the formation of tandem product (**C**). Thus, the optimum reaction condition for the nanocatalyst **5** to achieve the best tandem efficiency is $\hbox {Catalyst/4-bromoacetophenone/PhB(OH)}_{2}\hbox {/NaOH 1mg/0.1}$ mmol/0.14 mmol/0.7 mmol, $^{i}\hbox {PrOH}$ 1 mL and reaction time 6 h (entry 1, Table 1). The tandem reaction using nanocatalyst **3** and **4** were also conducted. Both the nanocatalyst **3** and **4** under identical reaction condition resulted in much inferior yield of tandem product **C** (25% and 15%) as compared to the mixed-valent nanocatalyst **5** (entries 7 and 8, Table 1) leaving a major amount of hydrogenated product **A** (65% and 76%, respectively). This implies the importance of the presence of palladium in both ($\hbox {Pd}^{\mathrm{II}}$ and $\hbox {Pd}^{0}$) oxidation states. It is also observed that pre-attainment of suitable oxidation states of metal atoms $[\hbox {Pd}^{0}$ and $\hbox {Pd}^{\mathrm{II}}]$ for two mechanistically different reactions leads to much higher catalytic efficiency than that of monovalent $[\hbox {Pd}^{0}$ or $\hbox {Pd}^{\mathrm{II}}]$ systems^27^. Both the nanocatalyst $\hbox {Fe}_{3}\hbox {O}_{4}\hbox {NP}$ (**1**) and $\hbox {Fe}_{3}\hbox {O}_{4}\hbox {@SiO}_{2}\hbox {NP}$ (**2**) without any $\hbox {Pd}^{\mathrm{II}}/\hbox {Pd}^{0}$ were unable to yield the tandem product (**A**). However, the nanocatalyst **1** was found to act as a good transfer hydrogenation catalyst (Yield of **A**, 90%), whereas, the nanocatalyst **2** exhibited poor yield of **A** (30%) using excess of catalyst (5 mg in each cases) and longer reaction time 18 h (entry 11 and 12, Table 1).

**Table 1:** Tandem Suzuki coupling/transfer hydrogenation of 4-bromoacetophenone and phenylboronic acid.


Entry	Cat.	ROH	Base	$\hbox {Yield}^{\mathrm{a}}$		
				A	B	C
1	**5**	$i\hbox {PrOH}$	NaOH	0	Trace	90
2	**5**	$i\hbox {PrOH}$	KOH	Trace	0	85
3	**5**	$i\hbox {PrOH}$	$\hbox {KO}^{t}\hbox {Bu}$	$20^{*}$	10	$56^{*}$
4	**5**	$i\hbox {PrOH}$	$\hbox {Cs}_{2}\hbox {CO}_{3}$	0	93	0
5	**5**	n-PrOH	NaOH	0	45	10
6	**5**	$\hbox {PhCH}_{2}\hbox {OH}$	NaOH	0	Trace	0
7	$\mathbf{5+Hg}$	iPrOH	NaOH	0	0	15
8^b^	**3+4**	iPrOH	NaOH	0	0	75
9^c^	**3**	$i\hbox {PrOH}$	NaOH	65	0	$25^{*}$
10^c^	**4**	$i\hbox {PrOH}$	NaOH	76	0	$15^{*}$
11^c,d^	**2**	$i\hbox {PrOH}$	NaOH	30	0	0
12^c,d^	**1**	$i\hbox {PrOH}$	NaOH	90	0	0

Reaction condition: $\hbox {Catalyst/4-bromoacetophenone/ PhB(OH)}_{2}/\hbox {base 1.0 mg/0.1}$ mmol/0.14 mmol/0.7 mmol, ROH 1.0 mL, reaction temperature $85\,^{{\circ }}\hbox {C}$, reaction time 6 h.

$^{\mathrm{a}}$Isolated yields ($^{*1}\hbox {H}$ NMR Yield).

$^{\mathrm{b}}$Amount of **3** and **4** is 1.0 mg each.

$$^{\mathrm{c}}$$Reaction time 18 h.

$^{\mathrm{d}}$Catalyst amount 5.0 mg.

**Figure 4:**
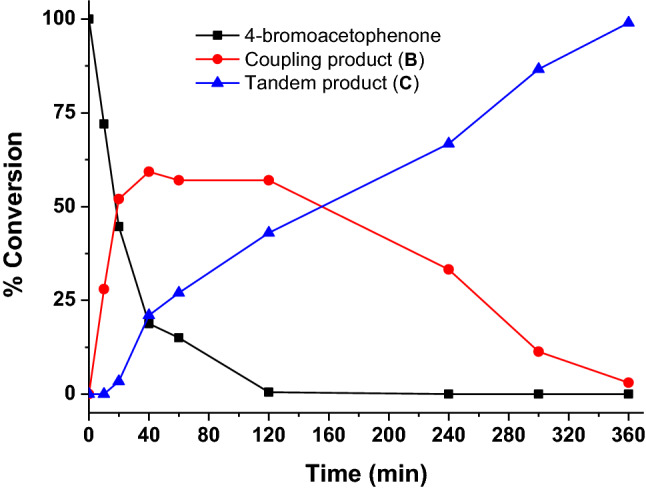
Reaction profile diagram for the tandem Suzuki coupling/transfer hydrogenation reaction catalyzed nanocomposite **5**.

Next, we conducted a time monitored reaction profile study of 4-bromoacetophenone and phenylboronic acid in the presence of NaOH in $^{i}\hbox {PrOH}$ at 85 $^{\mathrm{o}}\hbox {C}$ using nanocatalyst **5** (Fig. 4). It appears from the reaction profile diagram that the Suzuki coupling reaction and transfer hydrogenation reaction occur simultaneously, and the rate of C–C coupling reaction is faster than the transfer hydrogenation reaction (Fig. 4). In fact, the transfer hydrogenation reaction follows an induction period of 15 min. This suggests that the C–C coupling reaction is more facile than the transfer hydrogenation reaction under this set of reaction condition. The reaction profile study further demonstrates the consecutive nature of mechanistically independent reactions, where reaction initiated with C–C coupling reaction between 4-bromoacetophenone and phenylboronic acid in step I, and subsequently, the coupling product (**B**) gets hydrogenated to yield the tandem product (**C**) in step II. Palladium mediated C–C coupling reaction or transfer hydrogenation is known; however, tandem reaction utilizing mixed-valent palladium centers ($\hbox {Pd}^{0}/\hbox {Pd}^{\mathrm{II}}$) is rare^27,56,58^. Thus, the present ligand-free mixed-valent $\hbox {Pd}^{0}/\hbox {Pd}^{\mathrm{II}}$ nanocatalyst system **5** is an important addition to this tandem catalysis.

Further, the reversibility of the catalytic tandem Suzuki-Miyaura C–C coupling/transfer hydrogenation reaction was monitored using $^{1}\hbox {H}$ NMR spectroscopy by conducting the reverse reaction using the tandem product 1-([1,$1'$-biphenyl]-4-yl)ethan-1-ol (**C**) under the identical reaction condition. No trace of the progress of the reaction was observed in $^{1}\hbox {H}$ NMR spectrum, indicating irreversible nature of the catalytic cycle.

**Figure 5:**
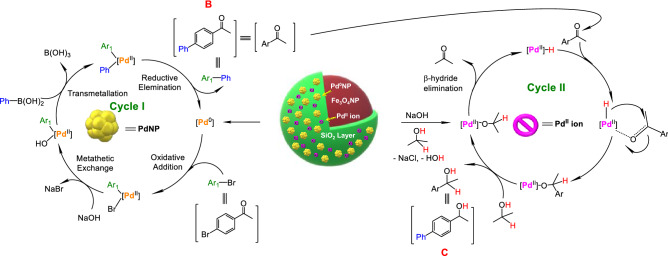
Plausible mechanism for tandem Suzuki coupling/transfer hydrogenation reaction catalysed by Fe_3_O_4_@SiO_2_/Pd^0^/Pd^II^NP (**5**) nanocomposite^59–63^.

Based on this experimental finding and literature data a plausible tandem reaction mechanism is proposed (Fig. 5)^59–63^. The tandem reaction is initiated with the formation of Suzuki-Miyaura C–C coupling reaction product (**B**) between 4-bromoacetophenone and phenylboronic acid at $\hbox {Pd}^{0}$ center (catalytic cycle I). The formed coupling product then enters into the catalytic cycle II, where it gets transfer hydrogenated to tandem product (**C**) catalyzed by $\hbox {Pd}^{\mathrm{II}}$ center in the presence of $^{i}\hbox {PrOH}$ as hydrogen donor.

Next, we extended our study to check the generality of the tandem Suzuki coupling/transfer hydrogenation reaction. The tandem reaction was conducted using different boronic acids of varying substituents (Table 2). In all the cases moderate to good yield of tandem product was registered. This suggest that the present nanocatalyst **5** has a good tolerance against various substrates. Further, the nature of substituents and their position in both boronic acid and haloarylketone are found to have a significant influence on the formation of tandem products.

Next, we performed mercury drop test to check the heterogeneity of the catalyst **5**^6,64,65^. The yield of tandem product **C** reduced significantly to 15% (entry 7, Table 1) upon addition of metallic mercury during the course of reaction. This suggests the heterogeneous nature of the nanocatalyst **5**. Further, to check the heterogeneity and leaching of palladium to the solution, hot filtration study was performed by following the reported procedure^66^. The filtrate did not show any catalytic activity under the optimized reaction condition. This further suggests that there is no leaching of the catalyst and the catalysis is heterogeneous in nature. Nevertheless, leaching of a small fraction of palladium from the nanocomposite surface to the solution may not be ruled out as there is formation of small amount of tandem product (**C**) during mercury poisoning test (entry 7, Table 1).

**Table 2:**
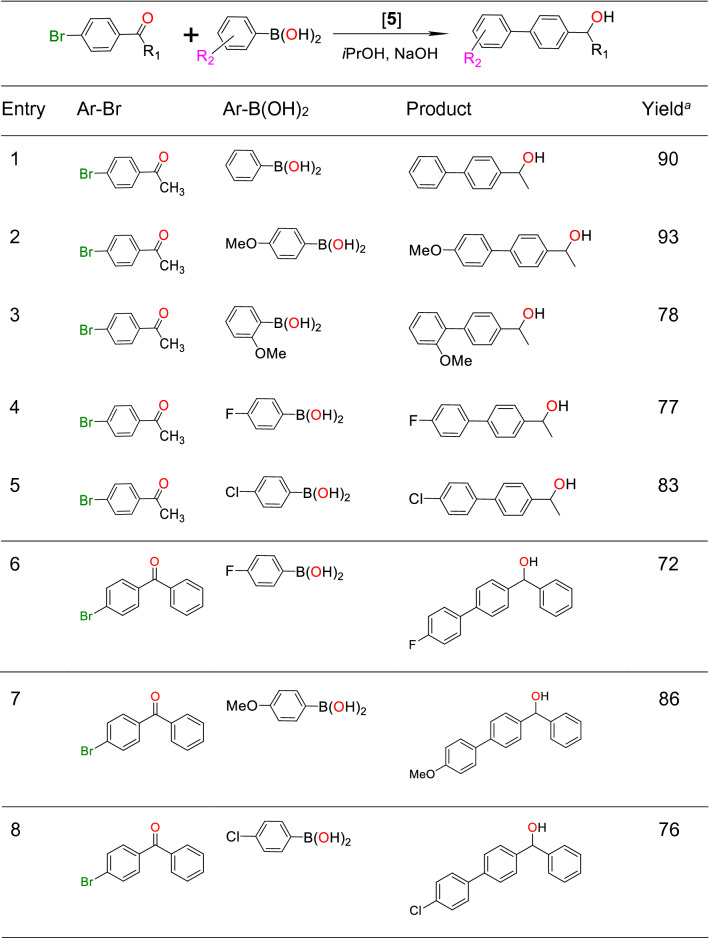
Tandem Suzuki coupling/transfer hydrogenation of 4-bromoacetophenone.

Reaction condition: Catalyst/4-bromoacetophenone/Ar-B(OH)_2_/NaOH 1 mg/0.1 mmol/0.14 mmol/0.7 mmol, ^*i*^PrOH 1 mL, reaction temperature 85 °C, reaction time 6 h. ^a^Isolated yields.

**Figure 6:**
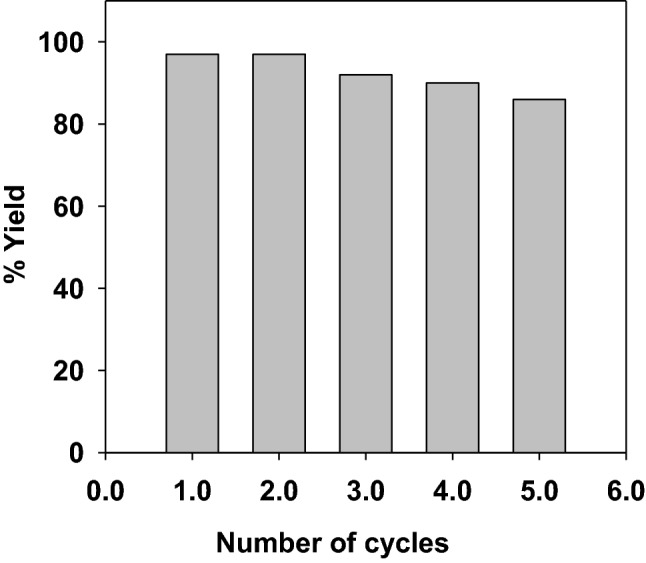
Reusability of Fe_3_O_4_@SiO_2_/Pd^0^/Pd^II^NP (**5**) nanocatalyst towards tandem Suzuki coupling/transfer hydrogenation reaction.

Another interesting feature of the present mixed-valent nanocatalyst Fe_3_O_4_@SiO_2_/Pd^0^/Pd^II^NP (**5**) is its separability and reusability from the solution as it consists of magnetic $\hbox {Fe}_{3}\hbox {O}_{4}$ core. The nanocatalyst **5** exhibited its facile separation ability from aqueous solution by using an external magnet during its preparation. To check the separability and reusability, the nanocatalyst **5** was magnetically retrieved from the reaction mixture after completion of the reaction. The magnetically separated nanocatalyst **5** was washed thoroughly with $^{i}\hbox {PrOH}$, dried, and reused for the tandem reaction. Interestingly, the tandem reactivity of nanocatalyst **5** did not change significantly, even after 5 cycles (Fig. 6). This demonstrates the efficient recyclability and reusability of nanocatalyst **5,** which is a desirable criterion for a good catalyst. Catalyst system exhibiting good tandem efficacy as well as excellent separation ability, and reusability are relatively rare. Thus, the present mixed-valent nanocatalyst **5** is an important example of tandem catalysis.

## Conclusions

In conclusion, we have developed a magnetically retrievable efficient mixed-valent nanocatalyst Fe_3_O_4_@SiO_2_/Pd^0^/Pd^II^NP (**5**). The nanocatalyst was characterized by FE-SEM-EDS, TEM, powder XRD techniques. The mixed-valent palladium ($\hbox {Pd}^{0}/\hbox {Pd}^{\mathrm{II}}$) was confirmed by XPS analysis. Unlike mono-valent $\hbox {Fe}_{3}\hbox {O}_{4}\hbox {@SiO}_{2}/\hbox {Pd}^{0}\hbox {NP}$ (**3**) and Fe_3_O_4_@SiO_2_/Pd^II^NP (**4**), the mixed-valent Fe_3_O_4_@SiO_2_/Pd^0^/Pd^II^NP (**5**) nanocatalyst exhibits excellent tandem Suzuki coupling/transfer hydrogenation reactivity. The mixed-valent nanocatalyst **5** also offers facile magnetic separation and its repeated use for several cycles. A catalyst exhibiting tandem reactivity along with facile separation ability is rare. Thus, we envision that the present ligand-free mixed-valent nanocatalyst system **5** is an important example that will be useful for the communities working in the area of tandem catalysis. conclusions section should come in this section at the end of the article, before the acknowledgements.

## Experimental

### Materials and methods

All chemicals were purchased from commercial sources and used as received. All glassware was cleaned using aqua regia, thoroughly washed with double distilled water, and rinsed with copious amount triple distilled water and dried in the oven. All reactions were carried out under an inert atmospheric condition using standard Schlenk techniques under the dinitrogen atmosphere unless and otherwise stated.

### Instrumentation

$^{1}\hbox {H}$ and $^{13}\hbox {C}$ NMR spectra were recorded using Bruker 400 NMR spectrometer using $\hbox {CDCl}_{3}$ as solvent. FE-SEM images and EDS analyses were recorded using Zeiss Merlin compact Microscope and Oxford instruments, respectively by drop casting the nanoparticles sample on carbon tape. HRTEM images were acquired using JEOL JEM 2100 electron microscope. Powder XRD analyses were carried out using a Bruker D8 Advance Diffractometer (Bruker AXS) with Cu $\hbox {K}_{\upalpha }$ radiation ($\uplambda = 1.54$ Å) over a $2\uptheta$ range of $10^{\circ }{-}110^{\circ }$ with a scanning rate of $40^{\circ }/\hbox {min}$. The samples for powder XRD was prepared by making a thin film of nanocomposites on glass slide.

### Synthesis of $\hbox {Fe}_{3}\hbox {O}_{4}$ NP (1)

The $\hbox {Fe}_{3}\hbox {O}_{4}$ nanoparticles was prepared by following the reported procedure^25^. Briefly, $\hbox {FeSO}_{4}{\cdot }7\hbox {H}_{2}\hbox {O}$ (278 mg, 1 mmol) and $\hbox {Fe}_{2}\hbox {(SO}_{4})_{3}$ (400 mg, 1 mmol) were dissolved in 30 mL of water (3:1). The pH of the solution was adjusted to 10.0 by adding a solution of $\hbox {NH}_{4}\hbox {OH}$ (25%) with stirring. The reaction mixture was then heated at $60\,^{{\circ }}\hbox {C}$ for 1 h with constant stirring to yield $\hbox {Fe}_{3}\hbox {O}_{4}\hbox {NP}$ (**1**). The reaction mixture was cooled to room temperature for further use.

### Synthesis of $\hbox {Fe}_{3}\hbox {O}_{4}\hbox {@SiO}_{2}$ NP (2)

The $\hbox {Fe}_{3}\hbox {O}_{4}\hbox {@SiO}_{2}$ nanoparticles was also prepared by following the reported procedure^25^. To the solution of in-situ generated $\hbox {Fe}_{3}\hbox {O}_{4}\hbox {NP}$ (**1**) at room temperature, tetraethylorthosilicate (TEOS) (1 mL, 4.48 mmol) was added with vigorous stirring. The stirring was continued for 18 h at room temperature. The formed $\hbox {Fe}_{3}\hbox {O}_{4}\hbox {@SiO}_{2}\hbox {NP}$ (**2**) was magnetically retrieved, washed several times with water and dried for further use (weight 500 mg).

### Synthesis of $\hbox {Fe}_{3}\hbox {O}_{4}\hbox {@SiO}_{2}/\hbox {Pd}^{0}$ NP (3)

To 30 mL aqueous suspension of $\hbox {Fe}_{3}\hbox {O}_{4}\hbox {@SiO}_{2}\hbox {NP}$ (**2**), an aqueous solution of $\hbox {K}_{2}[\hbox {PdCl}_{4}]$ (50 mg, 0.15 mmol) was added with continuous stirring. The stirring was continued for 6 h and cooled it in ice bath. To this cold solution, an aqueous solution of $\hbox {NaBH}_{4}$ (50 mg, 1.32 mmol) was added with stirring. The stirring was continued for an additional 12 h. The formed magnetic $\hbox {Fe}_{3}\hbox {O}_{4}\hbox {@SiO}_{2}/\hbox {Pd}^{0}\hbox {NP}$ (**3**) was then separated using an external magnet and washed several times with distilled water followed by ethanol and dried under vacuum and used for further studies.

### Synthesis of Fe_3_O_4_@SiO_2_/Pd^II^NP (4)

To the aqueous solution (pH 10) of $\hbox {Fe}_{3}\hbox {O}_{4}\hbox {@SiO}_{2}\hbox {NP}$ (**2**), an aqueous solution of $\hbox {K}_{2}[\hbox {PdCl}_{4}]$ (50 mg, 0.15 mmol) was added with continuous stirring. The stirring was continued for 18 h. It was then washed several times with distilled water followed by ethanol and dried under vacuum to yield Fe_3_O_4_@SiO_2_/Pd^II^NP (**4**).

### Synthesis of $\hbox {Fe}_{3}\hbox {O}_{4}\hbox {@SiO}_{2}/\hbox {Pd}^{0}/\hbox {Pd}^{\mathrm{II}}$ NP (5)

The $\hbox {Fe}_{3}\hbox {O}_{4}\hbox {@SiO}_{2}\hbox {@Pd}^{0}\hbox {NP}$ (**3**) was suspended in 30 mL distilled water, and 4 mL of ammonia solution was added to it to reach pH 10.0. To this solution, 50 mg (0.15 mmol) of $\hbox {K}_{2}[\hbox {PdCl}_{4}]$ was added and stirred for 18 h. The obtained nanoparticles was washed several times with distilled water followed by ethanol and dried under vacuum to yield $\hbox {Fe}_{3}\hbox {O}_{4}\hbox {@SiO}_{2}/\hbox {Pd}^{0}/\hbox {Pd}^{\mathrm{II}}$ (**5**) nanocomposite.

### Study of catalytic activity

In a 10 mL Schlenk tube 0.1 mmol of 4-bromoacetophenon and 0.14 mmol of phenylboronic acid, 0.7 mmol of NaOH and 1 mg of catalyst were taken. The reaction vessel was degassed and filled with dinitrogen by using standard Schlenk techniques. To this reaction mixture, 1 mL dry $^{i}\hbox {PrOH}$ was added. The dinitrogen environment of the reaction mixture was maintained by using a balloon filled with nitrogen gas. The reaction mixture was then heated at reflux for 6 h with continuous stirring in a preheated ($85\,^{\mathrm{o}}\hbox {C}$) oil bath. The reaction was quenched by adding 4 mL dichloromethane (DCM). The crude products were then charged in a silica gel column. The pure products were isolated either by column chromatography or preparatory thin layered chromatography by using 90% hexane and 10% ethyl acetate as eluent. The compounds were dried under vacuum, and isolated yields were calculated. $^{1}\hbox {H}$ NMR and $^{13}\hbox {C}$ NMR spectra of the dried products were recorded.

The identical reaction condition as above was maintained for plotting the reaction profile diagram. The reactions were quenched at different time intervals using DCM. Flash chromatography was carried out to remove the base and catalyst from the reaction mixture. The reaction mixture was then dried at reduced pressure. The composition of products in the reaction mixture at different time intervals was calculated using $^{1}\hbox {H}$ NMR spectroscopy by comparing the peak intensities of the compounds with the peak intensity of mesitylene (internal standard).

## Supplementary Information

Supplementary Figures.
