# Supplementary material for: A magnetically retrievable mixed-valent Fe3O4@SiO2/Pd0/PdII nanocomposite exhibiting facile tandem Suzuki coupling/transfer hydrogenation reaction
Source: Sci Rep. 2021 Apr 29;11:9305. doi: 10.1038/s41598-021-88528-6 (PMC8085233; doi:10.1038/s41598-021-88528-6)
Supplement: Supplementary file 1 — Supplementary Figures. [file 41598_2021_88528_MOESM1_ESM.docx]

A Magnetically Retrievable Mixed-valent Fe_3_O_4_@SiO_2_/Pd^0^/Pd^II^ Nanocomposite Exhibiting Facile Tandem Suzuki Coupling/Transfer Hydrogenation Reaction

Parminder Singh,^†,±^ Saumyaranjan Mishra,^†,±^ Anupam Sahoo^†^ and Srikanta Patra*^,†^

^†^School of Basic Sciences, Indian Institute of Technology Bhubaneswar, Argul, Jatni, Odisha-752050, India

^±^ Both the authors have contributed equally

**Table of content**

| **Sr. No.** | **Description** | **Page No.** |
| --- | --- | --- |
| 1 | **Scheme S1.** Synthetic scheme for the preparation of magnetically retrievable Fe_3_O_4_@SiO_2_@Pd^0^Pd^II^NP nanocatalytic system | 3 |
| 2 | **Figure S1**. Powder XRD spectra of nanoparticles (**1**-**5**) | 4 |
| 3 | **Figure S2.** FE-SEM and EDS images of Fe_3_O_4_NP (**1**), Fe_3_O_4_@SiO_2_NP (**2**); Fe_3_O_4_@SiO_2_/Pd^0^NP (**3**) and Fe_3_O_4_@SiO_2_/Pd^II^NP (**5**). | 5 |
| 4 | **Figure S3.** (a) FE-SEM, (b) EDS and (c) atom mapping images of Fe_3_O_4_@SiO_2_/Pd^0^/Pd^II^NP (**5**). | 6 |
| 5 | **Figure S4.** ^1^H NMR spectra of 1-(4-Biphenylyl)ethanol (400MHz, CDCl_3_, 298 K). | 7 |
| 6 | **Figure S5.** ^13^C NMR spectra of 1-(4-Biphenylyl)ethanol (400MHz, CDCl_3_, 298 K). | 7 |
| 7 | **Figure S6.** ^1^H NMR spectra of 1-(2^/^-methoxy-[1,1^/^-biphenyl]-4-yl)ethanol (400MHz, CDCl_3_, 298 K). | 8 |
| 8 | **Figure S7.** ^13^C NMR spectra of 1-(2^/^-methoxy-[1,1^/^-biphenyl]-4-yl)ethanol (400MHz, CDCl_3_, 298 K). | 8 |
| 9 | **Figure S8.** ^1^H NMR spectra of 1-(2^/^-methoxy-[1,1^/^-biphenyl]-4-yl)ethanol (400MHz, CDCl_3_, 298 K). | 9 |
| 10 | **Figure S9.** ^13^C NMR spectra of 1-(2^/^-methoxy-[1,1^/^-biphenyl]-4-yl)ethanol (400MHz, CDCl_3_, 298 K). | 9 |
| 11 | **Figure S10.** ^1^H NMR spectra of 1-(4^/^-fluro-[1,1^/^-biphenyl]-4-yl) ethanol (400MHz, CDCl_3_, 298 K). | 10 |
| 12 | **Figure S11.** ^13^C NMR spectra of 1-(4^/^-chloro-[1,1^/^-biphenyl]-4-yl) ethanol (400MHz, CDCl_3_, 298 K). | 10 |
| 13 | **Figure S12.** ^1^H NMR spectra of (4'-methoxy-[1,1'-biphenyl]-4-yl)(phenyl)methanol (400MHz, CDCl_3_, 298 K). | 11 |
| 14 | **Figure S13.** ^13^C NMR spectra of (4'-methoxy-[1,1'-biphenyl]-4-yl)(phenyl)methanol (400MHz, CDCl_3_, 298 K). | 11 |
| 15 | **Figure S14.** ^1^H NMR spectra of (4'-chloro-[1,1'-biphenyl]-4-yl)(phenyl)methanol (400MHz, CDCl_3_, 298 K). | 12 |
| 16 | **Figure S15.** ^13^C NMR spectra of (4'-chloro-[1,1'-biphenyl]-4-yl)(phenyl)methanol (400MHz, CDCl_3_, 298 K). | 12 |
| 17 | **Figure S16.** ^1^H NMR spectra of (4'-fluoro-[1,1'-biphenyl]-4-yl)(phenyl)methanol (400MHz, CDCl_3_, 298 K). | 13 |
| 14 | **Figure S17.** ^13^C NMR spectra of (4'-fluoro-[1,1'-biphenyl]-4-yl)(phenyl)methanol (400MHz, CDCl_3_, 298 K). | 13 |
| 15 | **Figure S18.** ^1^H NMR spectra of (1-(4-bromophenyl)ethan-1-ol (400MHz, CDCl_3_, 298 K). | 14 |
| 16 | **Figure S19.** ^13^C NMR spectra of (1-(4-bromophenyl)ethan-1-ol (400MHz, CDCl_3_, 298 K). | 14 |

**Scheme S1.** Synthetic scheme for the preparation of magnetically retrievable Fe_3_O_4_@SiO_2_/Pd^0^Pd^II^NP nanocatalytic system.

**Figure S1**. Powder XRD spectra of nanoparticles (**1**-**5**).

**Figure S2.** FE-SEM and EDS images of Fe_3_O_4_NP (**1**), Fe_3_O_4_@SiO_2_NP (**2**); Fe_3_O_4_@SiO_2_/Pd^0^NP (**3**) and Fe_3_O_4_@SiO_2_/Pd^II^NP (**4**).

**Figure S3.** (a) FE-SEM, (b) EDS and (c) atom mapping images of Fe_3_O_4_@SiO_2_/Pd^0^/Pd^II^NP (**5**).

**Figure S4.** ^1^H NMR spectra of 1-(4-Biphenylyl)ethanol (400MHz, CDCl_3_, 298 K).

**Figure S5.** ^13^C NMR spectra of 1-(4-Biphenylyl)ethanol (400MHz, CDCl_3_, 298 K).

**Figure S6.** ^1^H NMR spectra of 1-(2^/^-methoxy-[1,1^/^-biphenyl]-4-yl)ethanol (400MHz, CDCl_3_, 298 K).

**Figure S7.** ^13^C NMR spectra of 1-(2^/^-methoxy-[1,1^/^-biphenyl]-4-yl)ethanol (400MHz, CDCl_3_, 298 K).

**Figure S8.** ^1^H NMR spectra of 1-(2^/^-methoxy-[1,1^/^-biphenyl]-4-yl)ethanol (400MHz, CDCl_3_, 298 K).

**Figure S9.** ^13^C NMR spectra of 1-(2^/^-methoxy-[1,1^/^-biphenyl]-4-yl)ethanol (400MHz, CDCl_3_, 298 K).

**Figure S10.** ^1^H NMR spectra of 1-(4^/^-chloro-[1,1^/^-biphenyl]-4-yl) ethanol (400MHz, CDCl_3_, 298 K).

**Figure S11.** ^13^C NMR spectra of 1-(4^/^-chloro-[1,1^/^-biphenyl]-4-yl) ethanol (400MHz, CDCl_3_, 298 K).

**Figure S12.** ^1^H NMR spectra of (4'-methoxy-[1,1'-biphenyl]-4-yl)(phenyl)methanol (400MHz, CDCl_3_, 298 K).

**Figure S13.** ^13^C NMR spectra of (4'-methoxy-[1,1'-biphenyl]-4-yl)(phenyl)methanol (400MHz, CDCl_3_, 298 K).

**Figure S14.** ^1^H NMR spectra of (4'-chloro-[1,1'-biphenyl]-4-yl)(phenyl)methanol (400MHz, CDCl_3_, 298 K).

**Figure S15.** ^13^C NMR spectra of (4'-chloro-[1,1'-biphenyl]-4-yl)(phenyl)methanol (400MHz, CDCl_3_, 298 K).

**Figure S16.** ^1^H NMR spectra of (4'-fluoro-[1,1'-biphenyl]-4-yl)(phenyl)methanol (400MHz, CDCl_3_, 298 K).

**Figure S17.** ^13^C NMR spectra of (4'-fluoro-[1,1'-biphenyl]-4-yl)(phenyl)methanol (400MHz, CDCl_3_, 298 K).

**Figure S18.** ^1^H NMR spectra of 1-(4-bromophenyl)ethan-1-ol (400MHz, CDCl_3_, 298 K).

**Figure S19.** ^13^C NMR spectra of (1-(4-bromophenyl)ethan-1-ol (400MHz, CDCl_3_, 298 K).
